# Selective Increase of Correlated Activity in Arc-Positive Neurons after Chemically Induced Long-Term Potentiation in Cultured Hippocampal Neurons

**DOI:** 10.1523/ENEURO.0540-20.2021

**Published:** 2021-12-01

**Authors:** Yuheng Jiang, Antonius M. J. VanDongen

**Affiliations:** Program for Neuroscience and Behavioral Disorders, Duke-NUS Medical School, 169857 Singapore

**Keywords:** Arc, engram, LTP, memory, network, plasticity

## Abstract

The activity-dependent expression of immediate-early genes (IEGs) has been utilized to label memory traces. However, their roles in engram specification are incompletely understood. Outstanding questions remain as to whether expression of IEGs can interplay with network properties such as functional connectivity and also if neurons expressing different IEGs are functionally distinct. In order to connect IEG expression at the cellular level with changes in functional connectivity, we investigated the expression of 2 IEGs, Arc and c-Fos, in cultured hippocampal neurons. Primary neuronal cultures were treated with a chemical cocktail [4-aminopyridine (4AP), bicuculline (Bic), and forskolin] to increase neuronal activity, IEG expression, and induce chemical long-term potentiation (LTP). Neuronal firing is assayed by intracellular calcium imaging using GCaMP6m and expression of IEGs is assessed by immunofluorescence staining. We noted an emergent network property of refinement in network activity, characterized by a global downregulation of correlated activity, together with an increase in correlated activity between subsets of specific neurons. Subsequently, we show that Arc expression correlates with the effects of refinement, as the increase in correlated activity occurs specifically between Arc-positive neurons. The expression patterns of the IEGs c-Fos and Arc strongly overlap, but Arc was more selectively expressed than c-Fos. A subpopulation of neurons positive for both Arc and c-Fos shows increased correlated activity, while correlated firing between Arc+/cFos– neurons is reduced. Our results relate neuronal activity-dependent expression of the IEGs Arc and c-Fos on the individual cellular level to changes in correlated activity of the neuronal network.

## Significance Statement

Establishing a stable long-lasting memory requires neuronal network-level changes in connection strengths in a subset of neurons, which together constitute a memory trace or engram. Two genes, c-Fos and Arc, have been implicated to play critical roles in the formation of the engram. They have been studied extensively at the cellular/molecular level and have been used as markers of memory traces in mice. We have correlated Arc and c-Fos cellular expression with refinement of correlated neuronal activity following pharmacological activation of networks formed by cultured hippocampal neurons. Whereas there is a global loss of correlated activity, Arc-positive neurons show selectively increased correlated activity. Arc is more selectively expressed than c-Fos, but the two genes act together in encoding information about changes in correlated firing.

## Introduction

Activity-driven neuronal plasticity is the basis for learning and memory. New tools in optogenetics and molecular biology have culminated in numerous studies in recent years which mark immediate-early gene (IEG)-expressing neurons as memory engrams (Liu et al., 2012; Denny et al., 2014; Tonegawa et al., 2015). Transcriptional programs activated on neuronal stimulation are complex, with the involvement of a myriad of IEGs, their downstream effectors and the interplay between gene transcription and ongoing neuronal activity (Yap and Greenberg, 2018). Although the activity-dependent expression of IEGs has been successfully utilized to label memory traces, their roles in engram specification is incompletely understood. For instance, although the expression profiles of IEGs within neuronal populations overlap significantly (Mahringer et al., 2019), they are diverse and distinct (Gonzales et al., 2019), with different IEGs performing distinct functions. Furthermore, it is known that within single neurons, there is a combinatorial expression of IEGs (Sheng et al., 1995). This suggests that differential expression of IEGs can encode the specificity of the stimulus, which has been demonstrated in part using single-cell RNA-sequencing technology (Jaeger et al., 2018). Activity-regulated cytoskeleton-associated protein/activity-regulated gene 3.1 (Arc/Arg3.1) and c-Fos are two IEGs that are commonly used in memory engram labeling experiments (Kawashima et al., 2014). Arc is an important player in synaptic plasticity and long-term memory consolidation (Plath et al., 2006; Shepherd and Bear, 2011; Nikolaienko et al., 2018). c-Fos is another IEG whose increase in expression is commonly used as an indicator for neuronal activation. It was one of the first transcription factors whose induction was shown to be activity dependent (Sheng and Greenberg, 1990). It has been recently shown that IEGs Arc and c-Fos are differentially expressed within neuronal populations and their combinatorial expression is functionally linked to whether a memory trace is newly formed or reactivated (Jaeger et al., 2018). This is because of the difference in the expression dynamics of the two IEGs, with c-Fos being transiently expressed after activation but Arc maintaining a high level of transcription hours after stimulus onset, specifically in the dentate granule neurons (Jaeger et al., 2018). Further questions remain, however, as to whether expression of IEGs can interplay with patterns of correlated firing between neurons and if neurons expressing different IEGs are functionally distinct.

The fundamental cellular correlate of memory is synaptic plasticity, while engram formation is dependent on the connectivity of the neuronal network (Tonegawa et al., 2015). Conventionally, studies of neuronal activity and IEG expression are mostly based on the single cell level whereas network activity is recorded on the neuronal population level. Thus, there is a lack in linking IEG expression and changes in patterns of correlated activity changes on the network level. Similarly, studies on long-term potentiation (LTP) are extensive on the molecular, cellular and pathway level, but few studies have focused on the effects of LTP in altering patterns of correlated activity in a population of interconnected neurons. Among the various induction methods of LTP, direct chemical stimulation of specific biochemical processes to induce LTP can result in plasticity changes in large numbers of synapses from multiple neurons. NMDA receptor-dependent LTP requires the coincidence of presynaptic glutamate release and postsynaptic depolarization. Chemically, this can be accomplished by a variety of pharmacological agents. Among these, bicuculline (Bic), a GABA_A_ receptor antagonist has been shown to be able to induce bursts of action potential and elicit NMDA receptor-dependent calcium transients. When this is applied together with the presynaptic K^+^ channel blocker 4-aminopyridine (4AP), which enhances glutamate release, calcium transients in the neuronal nucleus increase from baseline levels (Hardingham et al., 2001). This sustained elevation in nuclear calcium can increase the affinity of calmodulin binding to calcium and in turn result in maintained activation of Ca^2+^/calmodulin-dependent enzymes that potentially facilitate transcription. The synergistic effects of these agents have been shown to be able to increase the endogenous expression of c-Fos (Hardingham et al., 2001). Forskolin, an adenylyl cyclase activator, can effectively increase the level of cAMP levels and directly activate transcription and translation that is important for the expression and maintenance of protein synthesis-dependent late phase of LTP (Otmakhov et al., 2004). It has also been shown that the translational repression of the IEG Arc is alleviated by application of forskolin, through activation of the cAMP-dependent protein kinase (PKA) pathway (Bloomer et al., 2008). This combination of pharmacological agents, namely 4AP, Bic, and forskolin, has been used effectively to induce LTP in cultured hippocampal neurons in the study of Arc dynamics (Wee et al., 2014; Oey et al., 2015; Leung et al., 2019).

We aim to relate neuronal activity-dependent expression of the IEGs Arc and c-Fos on the individual cellular level to changes in correlated activity in the cultured neuronal network. Changes in functional network connectivity can be studied systematically (Poli et al., 2016), and these methods can be applied to cultured neuronal networks. We made use of intracellular calcium imaging (GCaMP6m) as a proxy measurement for neuronal activity changes. Expression of IEGs Arc and c-Fos is assessed by immunofluorescence staining and is subsequently matched with changes in correlated activity in specific subpopulations of neurons.

## Materials and Methods

### Neuronal cell culture

Hippocampi from embryonic day 18 Sprague Dawley rats of either sex was dissected in ice-cold HBSS and digested using a papain dissociation system (Worthington Biochemical Corporation). The density of the network was determined by a seeding density of 1.5 × 10^5^ cells/cm^2^. Neurons was plated on glass-bottom culture dishes (MatTek) that had been coated with poly-D-lysine at 0.1 mg/ml (Invitrogen) for 2 h at 37°C. Neurons were cultured in Neurobasal medium supplemented with 2% B-27, 0.5 mm L-glutamine, 10% penicillin/streptomycin, and half of the medium is replaced bi-weekly from day *in vitro* (DIV)5 onwards. Experiments were conducted from DIV20 to DIV23. All animal procedures were performed in accordance with the Institutional Animal Care and Use Committee (IACUC) regulations.

### Neuronal stimulations and silencing

We used a combination of 4AP, Bic, and forskolin at final concentrations of 100, 50, and 50 μm, respectively (4BF) to stimulate synaptic NMDA receptors and network activity and chemically induce LTP. The drugs were added to the culture medium for 2–4 h. Activity silencing was done by adding tetrodotoxin (TTX) at a concentration of 1 μm to the culture medium before treating the culture with the stimulation drugs.

### Conventional immunofluorescence

Cells were fixed with a solution containing 4% paraformaldehyde (PFA), 4% sucrose, and PBS for 15 min at room temperature (RT), blocked with a solution containing 10% goat serum, 2% bovine serum albumin (BSA), and PBS for 1 h at RT. Rabbit-anti-NeuN (Thermo Fisher Scientific catalog #711054, RRID:AB_2610583), mouse-anti-Arc (C7; Santa Cruz Biotechnology catalog #sc-17839, RRID: AB_626696), and rabbit-anti-c-Fos (Santa Cruz Biotechnology catalog #sc-52, RRID:AB_2106783) primary antibody were used at 1:300. Primary antibodies were incubated for 1 h at RT in a dilution buffer containing 1:1 block solution and PBS-Triton X-100 solution at 1:300. Dishes were washed three times with PBS-Triton X-100 and incubated with Alexa Fluor 488-conjugated secondary antibody 1:1000 (Invitrogen-Invitrogen) in dilution buffer for 1 h at RT. Washing was repeated as per the above. The cells were subsequently incubated with 5 μm DAPI for 5 min before being imaged.

### Transduction

For calcium imaging, the GCaMP6m construct (pAAV.Syn.GCaMP6m.WPRE.SV40) was a gift from Douglas Kim and GENIE Project (Addgene viral prep #100841-AAV9; http://n2t.net/addgene:100841; RRID:Addgene_100841), and transferred from University of Pennsylvania, Vector Core. It was transduced into the cells at a MOI of 1 × 10^5^ on DIV8. Half of the medium was changed on DIV9 to prevent virus toxicity.

### Widefield imaging

Fluorescence images were obtained using a motorized inverted wide-field epifluorescence microscope (Nikon Eclipse Ti-E), using Nikon 10× Plan Apo objective (N.A. = 0.4). Motorized excitation and emission filter wheels (Ludl Electronics) fitted with a DAPI/CFP/YFP/DsRed quad filter set (#86010, Chroma) were used together with filter cubes for DAPI, YFP, and TxRed (Chroma) to select specific fluorescence signals.

### Calcium imaging

**Figure 1.**
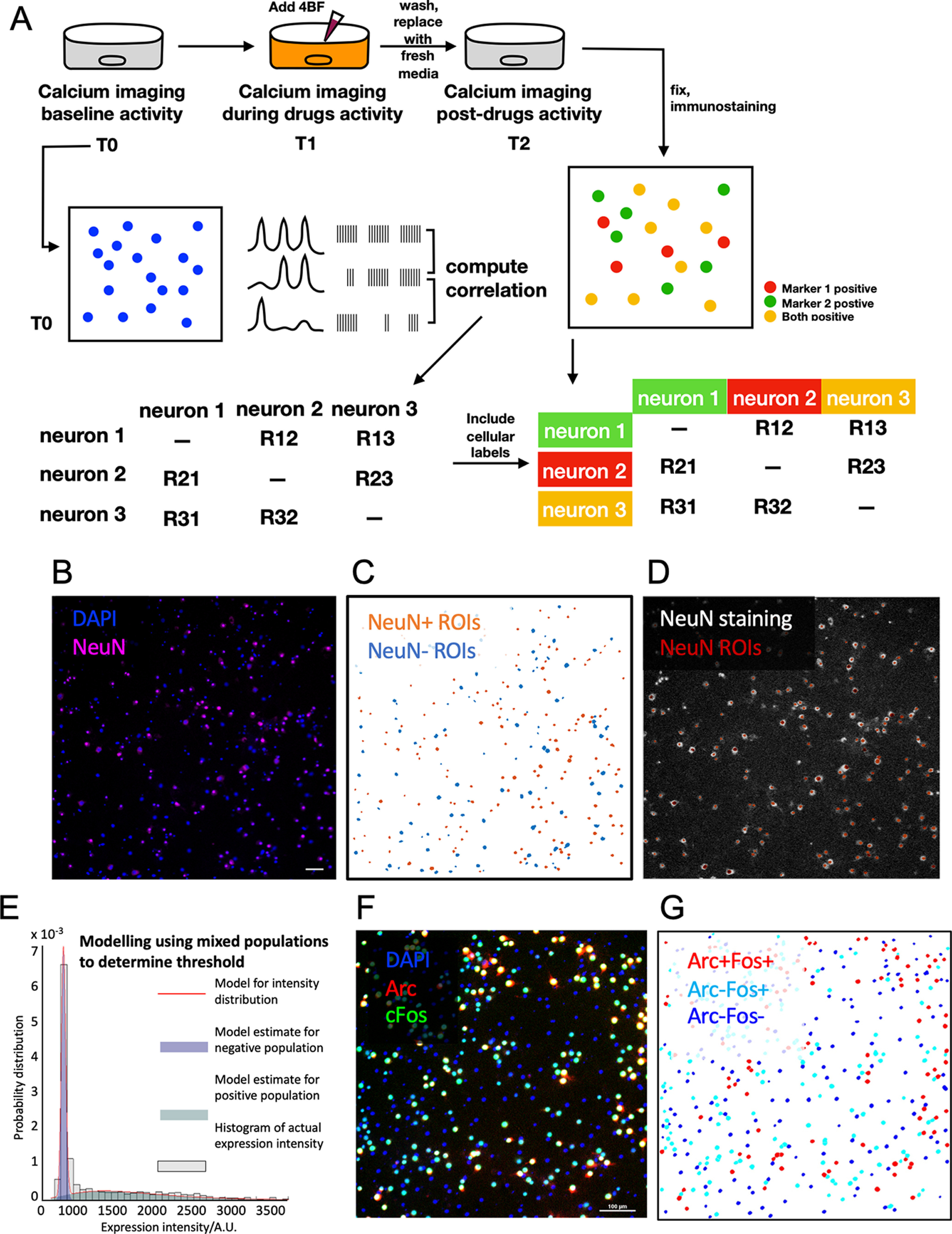
Calcium imaging pipeline and data analysis. ***A***, Schematic of calcium imaging: cells transduced with GCaMP6 are imaged under widefield epifluorescence imaging at baseline and after 4BF treatment. Calcium movies are processed to extract the calcium imaging data of individual neurons, which are converted into spike times using methods described. The correlation between neuronal spike times is calculated using the STTC. Cells were fixed and stained with the desired markers (NeuN with Arc or Arc with c-Fos). These were then used as identity markers for subsequent connectivity analysis. ***B***, Representative immunofluorescence staining image showing NeuN and DAPI. Extraction of calcium waves of individual neurons are based on selection of ROIs by segmentation of the DAPI signals. The intensity of NeuN staining was determined within each DAPI ROI. These expression values were modelled as two separate normal distributions (similar to panel ***E***), and the threshold for selection of positive populations was determined based on the distributions as described in Materials and Methods. ***C***, Following thresholding, the NeuN-positive population from each network can be labeled as shown, based on ROIs from DAPI segmentation. ***D***, The accuracy of thresholding can be verified visually by overlaying the NeuN-positive ROIs onto immunostaining images of NeuN. ***E***, Threshold selection was done based on mixed population modeling. Values of expression intensities was plotted according to their probability distributions and modelled as two normal distributions. The mean and SDs of these two distributions were obtained computationally and threshold was selected based on these values as described in Materials and Methods. This method was also used to determine the threshold for Arc-positive and c-Fos-positive populations. ***F***, Representative immunofluorescence staining image showing Arc and c-Fos with DAPI. ***G***, Representative post-analysis reconstruction image indicating ROI determination by DAPI and allocation of ROIs according to Arc and c-Fos identity as determined by their expression levels.

Calcium imaging using widefield microscopy was done on neuronal cultures transduced with GCaMP6. Movies of 1-min duration were taken at a frame rate of 5 Hz at baseline, after 4BF treatment and after 4BF removal. Upon completion of calcium imaging, the culture was fixed and stained as described. Figure 1*A* shows the calcium imaging and analysis pipeline.

### Data analysis and graphs

Graphs were plotted using either GraphPad Prism Software or with MATLAB (version R2017b). Data are presented as mean with SEM. All custom-written codes for MATLAB used for the following analyses are available on GitHub at https://github.com/yuhengj/network_arc_dynamics.

### Threshold selection

The determination of threshold for selection of positive populations was done systematically with custom-written MATLAB codes, which model the expression values as two separate normal distributions (an example of this is shown in Fig. 1*E*). As there is some overlap of the negative and positive populations, we aimed to reduce false positives by setting the threshold for positive cells to be at values higher than 99.7% (three times SD) of the negative population.

### Calcium transient and spike detection

Images were acquired with the Nikon NIS Elements software and subsequently processed with MATLAB, with the BioFormats plugin. Calcium transients from the whole network were computed based on the total fluorescence change over time. Photobleaching was accounted for with fluorescence intensity from cells that were silent during the recording (lowest tenth percentile of the whole population of cells). Peaks in fluorescence were detected using MATLAB function *findpeaks*. To obtain calcium signals from only the neuronal population, regions of interest (ROIs) were first determined from the DAPI images, and the expression levels of NeuN within the ROIs were quantified (Fig. 1*B*). NeuN-positive threshold was determined by mixed population modeling with expression levels from all cells (Fig. 1*E*), and the threshold for negative population was selected to be 3 SDs from the population mean. These NeuN-positive ROIs are then used to extract fluorescence signals from the calcium movies recorded. All pixels within each ROI are averaged to give a single time course, and calcium transients (ΔF/F) were calculated by subtracting each value with the mean of signals from the lowest 10% of neurons at each time point. This removes background and corrects for photobleaching over time. Spike times were then inferred from ΔF/F using a maximum a posteriori (MAP) estimate of the spike train developed by Joshua Vogelstein (Vogelstein et al., 2010) named fast-oopsi. This implementation was adapted to be used in MATLAB as part of the FluoroSNNAP package developed by Patel and colleagues (Patel et al., 2015), which is available for download at https://www.seas.upenn.edu/∼molneuro/software.html. Firing rates of neurons were calculated as the number of spikes divided by the total recording time.

### Neuronal activity correlations

Neuronal correlations were calculated using the spike time tiling coefficient (STTC) that was previously described (Cutts and Eglen, 2014). This was chosen as this measure is known to be independent of firing rate. Specifically, the correlation index (CI) is calculated according to Equation 1 below:

(1) $$\text{STTC }=\text{ 1}/\text{2}*(\left({\text{P}}_{\text{A}}-{\text{T}}_{\text{B}}\right)/\left(\text{1}-{\text{P}}_{\text{A}}{\text{T}}_{\text{B}}\right)+\left({\text{P}}_{\text{B}}-{\text{T}}_{\text{A}}\right)/(\text{1}-{\text{P}}_{\text{B}}{\text{T}}_{\text{A}})),$$

where T_A_ is the proportion of total recording time which lies within plus minus δ t of any spike from spike times of A. P_A_ is the proportion of spikes from A which lie within a small time window (±Δt) of any spike from B. Δt was set to 50 ms according to the previous study (Cutts and Eglen, 2014). T_B_ and P_B_ are calculated correspondingly, for any two given spike trains A and B. A maximum value of +1 can be obtained for complete correlation. This pair-wise synchronization index for each neuron can be used to populate a adjacency matrix M of size *n* × *n*, where *n* = number of neurons in the network. We used functions from the Brain Connectivity Toolbox (available for download at https://sites.google.com/site/bctnet) to edit the adjacency matrix.

### Change in neuronal correlations

To determine the change in neuronal correlations of the whole network, we borrowed a measure developed for functional MRI that can represent all the connection strengths in the network, termed the Intrinsic Connectivity Distribution (ICD; Scheinost et al., 2012). This metric allows all of the neuronal correlations in the network to be captured with only a few parameters and eliminates the need for setting arbitrary thresholds. ICD models the distribution of all the correlations within the network first as a degree metric, from which a survival curve can be obtained and modelled as a stretched exponential with variance parameter (α) and shape parameter (β) according to Equation 2:

(2) $$\text{d}\left(\tau ,\text{ x},\;\alpha \left(\text{x}\right),\;\beta \left(\text{x}\right)\right)={\text{e}}^{-\alpha \left(\text{x}\right)\tau \beta (\text{x})}.$$

These parameters allow for a comparison of the distribution of correlations within a network, without the need for setting any arbitrary threshold for correlation and enables comparison of a network across different time points.

### Firing rate analysis

The firing rates of individual neurons were calculated from NeuN-positive DAPI ROIs and separated into Arc-positive and Arc-negative values. The average firing rate at baseline was obtained for each culture by summing firing rate of individual neurons and dividing by the number of Arc-positive or Arc-negative neurons. The normalized change in firing rate during and after 4BF treatment was calculated by taking the change in firing as compared with baseline levels for each individual neuron and normalizing it to the firing rate at baseline also for each individual neuron. The average value for each network (total of *n* = 17) was then computed by taking the mean.

### Linear regression model for Arc-positive percentage

A stepwise linear regression model was used to fit the Arc-positive percentages data. The *stepwiselm* function from MATLAB was used to find optimal terms from a list of firing rates (initial, final, and change), CI (initial, final, and change), refinement status (binary) to explain the percentage of Arc. Analysis of variance of the linear model obtained was also conducted in MATLAB.

### Increases and decreases in CI

Measures of STTC changes were used to determine changes in CI between different pairs of neurons after 4BF treatment. Correlations that initially had low CI which later became high are called “positive-change correlations,” whereas correlations initially having high CI but later having low CI were referred to as “negative-change correlations.” Specifically, positive-change correlations were defined as initial CI between the values of 0 and 0.5 and final CI between 0.5 and 1 while negative-change correlations are defined as initial CI between 0 and 0.5 and have final CI values between 0.5 and 1. The corresponding value of the percentage of Arc-positive to Arc-positive correlations is determined by counting the numbers of Arc-Arc correlations over the total number of correlations at each correlation threshold. For positive-change correlations, the threshold is based on the final CI while for negative-change correlations the thresholds are determined based on the initial CI.

### Distance measure

Each neuronal nuclear ROI from which the immunofluorescent signal and calcium signal is extracted from, has a central coordinate (*x* and *y* coordinates based on pixels). Each type of connection between neuronal pairs therefore corresponds to a physical Euclidean distance that can be calculated based on the central coordinate. Each image frame was 1007 × 1007 pixels, with each pixel corresponding to 0.8 μm, giving a total area of 0.64 mm^2^.

## Results

To achieve LTP in cultured hippocampal neurons, we made use of a combination of 4AP, a blocker of presynaptic K_v_1 family K^+^ channels, Bic, a GABA receptor antagonist, together with forskolin, an adenylyl cyclase activator which enhances NMDA-dependent LTP (Otmakhov et al., 2004). This cocktail, subsequently referred to as 4BF, increased synchronized network bursting in hippocampal cultures. Calcium imaging and analysis were conducted as described in Materials and Methods and outlined in Figure 1*A*.

### 4BF increases neuronal firing rate but decreases overall neuronal activity correlations

**Figure 2.**
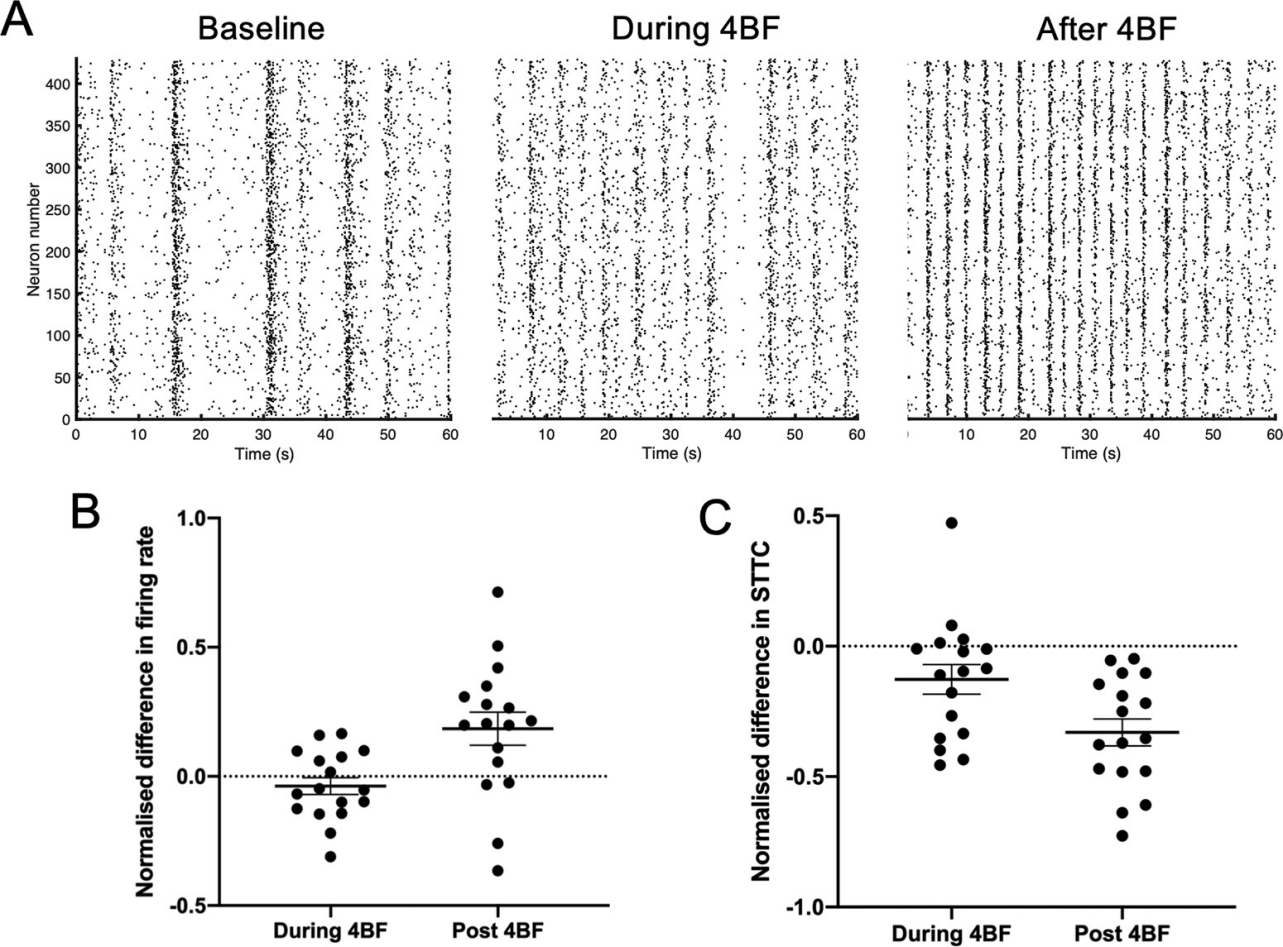
Neurons exhibit increased firing rate but decreased CI after 4BF treatment. ***A***, Representative raster plots of a culture at baseline and 3 h after being treated with 4BF. ***B***, Quantification of firing rates during and after 4BF treatment, both are compared and normalized to firing rates for each culture at baseline. Normalized difference in firing rate during 4BF = −0.0373, SEM = 0.0326. Normalized difference in firing rate after 4BF = 0.185, SEM = 0.0639; *n* = 17 for both. ***C***, Quantification of STTC during and after 4BF treatment, both are compared and normalized to the STTC for each culture at baseline. Normalized difference in STTC during 4BF = −0.127, SEM = 0.0569. Normalized difference in STTC after 4BF = −0.330, SEM = 0.0518; *n* = 17 for both. Firing rates were quantified from cultures that were subsequently stained with NeuN marker (*n* = 17 cultures), and only neuronal cells that are positive for NeuN were selectively used for quantification of firing rates.

**Figure 3.**
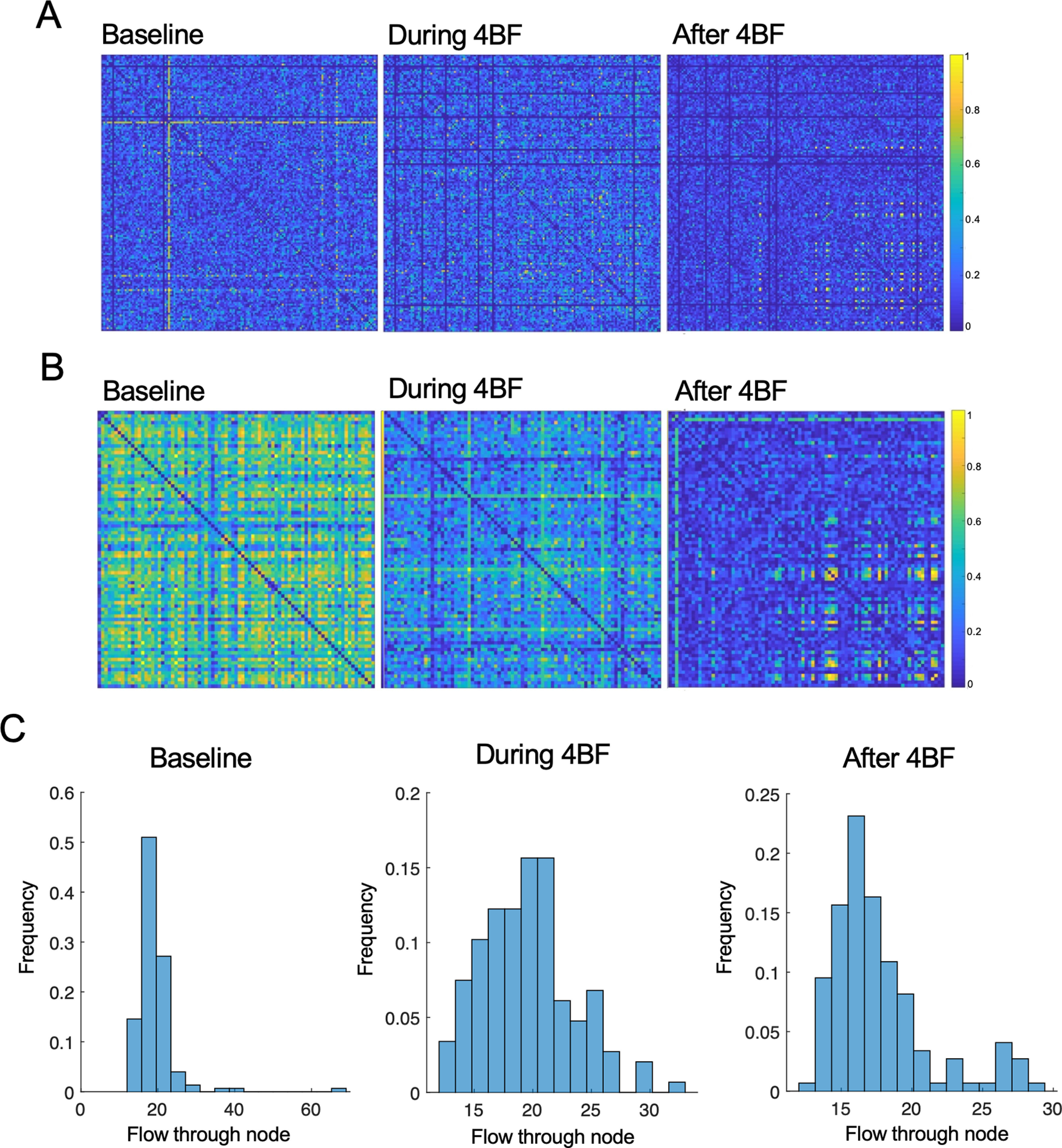
Refinement of correlated activity after 4BF. ***A***, A culture showing an initial state of low CI, with refinement after 4BF. ***B***, A culture showing an initial state of high CI with refinement after 4BF. ***C***, Histogram of total flow (sum of all CI through each node) at baseline, and during and after 4BF for a representative culture. There is a right shift in the distribution with a higher proportion of CI in the culture being of higher value after 4BF.

To assess the effects of 4BF on individual neurons, calcium traces for single neurons were extracted from the recordings. A representative raster plot of neuronal firing activity at baseline, during 4BF treatment and after 4BF treatment can be seen in Figure 2*A*. We found that there was a significant increase in the firing rate in neurons following 4BF treatment (Fig. 2*A*,*B*). To determine the index of correlated activity between different neurons in the culture, the STTC was used, and the CI between all neuronal pairs were obtained (Cutts and Eglen, 2014). This revealed differences in initial culture states, with ∼30% of the cultures (*n* = 8 out of total 28) having an overall high CI before the treatment by 4BF and the remaining dishes being low in neuronal correlation initially. Representative images of the correlations between neurons are represented as heatmaps in Figure 3*A*,*B*, with an overall high CI initial state (Fig. 3*B*, first panel) or a low CI initial state (Fig. 3*A*, first panel). The average CI for each network was found to be decreased as compared with baseline (Fig. 2*C*), demonstrating an overall decrease in neuronal activity correlations after 4BF.

### Refinement of network neuronal correlations by 4BF

To further investigate the distribution of activity correlations in the neuronal cultures, we examined the CI of each neuronal pair from the adjacency matrix and found that although there was a general reduction in CI after 4BF (Fig. 2*C*), some cultures had subsets of neurons that had a high CI after 4BF. This phenomenon was termed as refinement. Refinement happened independent of initial states of the network, in both cultures with high initial CI as well as networks with low initial CI (Fig. 3*A*,*B*). This refinement effect can also be visualized in the histogram of total flow (sum of all CIs through each node). As seen in Figure 3*C*, there is a small peak at the region of high CI (marked out with the red box). Refinement after 4BF was observed in 60% of the cultures (*n* = 10 out of a total 17).

**Figure 4.**
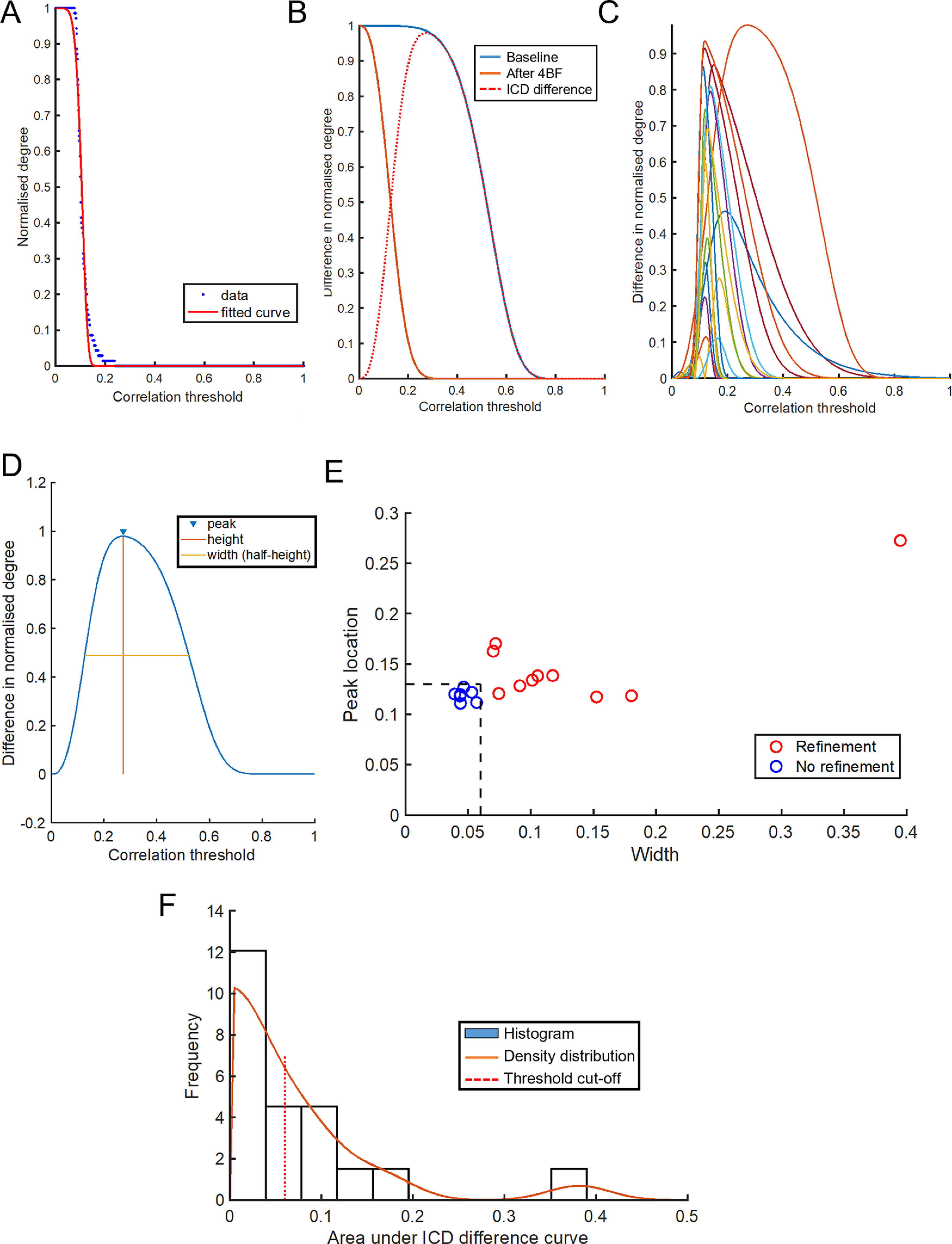
The ICD measure for describing neuronal correlations. ***A***, Survival curve obtained by thresholding the degree distribution at different values (*x*-axis values). The values above the threshold degree were integrated to obtain the normalized degree (*y*-axis values). Data at different values shown in blue, modeling of stretched exponential with variance (α) and parameter (β; Eq. 2; Materials and Methods), shown in red as the fitted curve. ***B***, The ICD distribution at baseline (blue) and after 4BF treatment (orange), and the ICD difference plot (red dotted line) for a sample culture. ***C***, ICD different plots for all cultures with each color representing a different culture. The width, peak height, and peak location of these curves can be obtained. ***D***, Different measures that can be obtained from the ICD difference plot: width of half-peak height, height, and location of peaks. ***E***, Cultures that have not undergone refinement have low peak widths and peak locations (marked in blue “o”), *n* = 7. Cultures that have undergone refinement have large peak widths and peak locations (marked in red “o”), *n* = 10. Refinement was verified visually based on the connectivity matrices. ***F***, The area under the ICD difference curves was also computed for each culture, and the distribution of the areas is plotted. The effects of refinement correlate with larger area under the ICD difference curves. However, this measure failed to capture two of the refined cultures (out of a total of 10).

To better quantify this refinement in network neuronal correlations, it is of interest to specifically identify the neurons exhibiting high CI. However, interculture comparisons may not be ideal, as the threshold for CI can vary among cultures. Using a standardized threshold for all cultures to determine the refinement effect is therefore not ideal. To remedy this problem, we employed a previously developed measure named ICD (Scheinost et al., 2012), which eliminates the need for setting arbitrary thresholds, and allows all of the correlation information to be captured with only a few parameters. Figure 3*C* shows the degree distribution of CIs as histograms, before, during, and after treatment with 4BF. Each degree distribution can give rise to a survival curve (Fig. 4*A*) by thresholding the degree distribution at different values (*x*-axis values). The values above the threshold degree were then integrated to obtain the normalized degree (*y*-axis values). These data were modelled as a stretched exponential according to Equation 2. The variance (α) and parameter (β) can be obtained from the estimated function (Fig. 4*A*). These parameters allow for a quantification of the CI distribution within each network, before and after 4BF treatment (Fig. 4*B*). An ICD difference plot can be obtained by subtraction of the before and after plots and reveals changes in CI distribution (Fig. 4*B*). This was then utilized to effectively compare the correlations within the network before and after 4BF treatment. The ICD difference curves for all the cultures examined can be seen in Figure 4*C*. Quantification of these curves was subsequently performed based on the width of half-peak height, the height and location of the peaks (Fig. 4*D*). For instance, a peak in ICD difference plot that is at a larger value of threshold indicates that there is a big change in CI before and after 4BF, and the width of half-peak height in the ICD difference plots indicates how widespread the change in CI is. For refinement, there is not only a global reduction in CI (corresponding to a larger threshold value at the peak) but also a wider distribution for the difference in CI (larger width of the ICD difference plots). The width measure and the peak location of the ICD difference plots were found to correlate well to whether the network has undergone refinement (Fig. 4*E*). The area under the ICD difference curves for all cultures were also obtained as a separate measure and it was found that larger area under the curve of ICD difference tended to correlate with effect of refinement (Fig. 4*F*). However, this measure failed to capture two (out of 21) of the refined networks. The effect of refinement was verified visually using the adjacency matrices.

### Arc expression correlates with the final network CI and refinement

**Figure 5.**
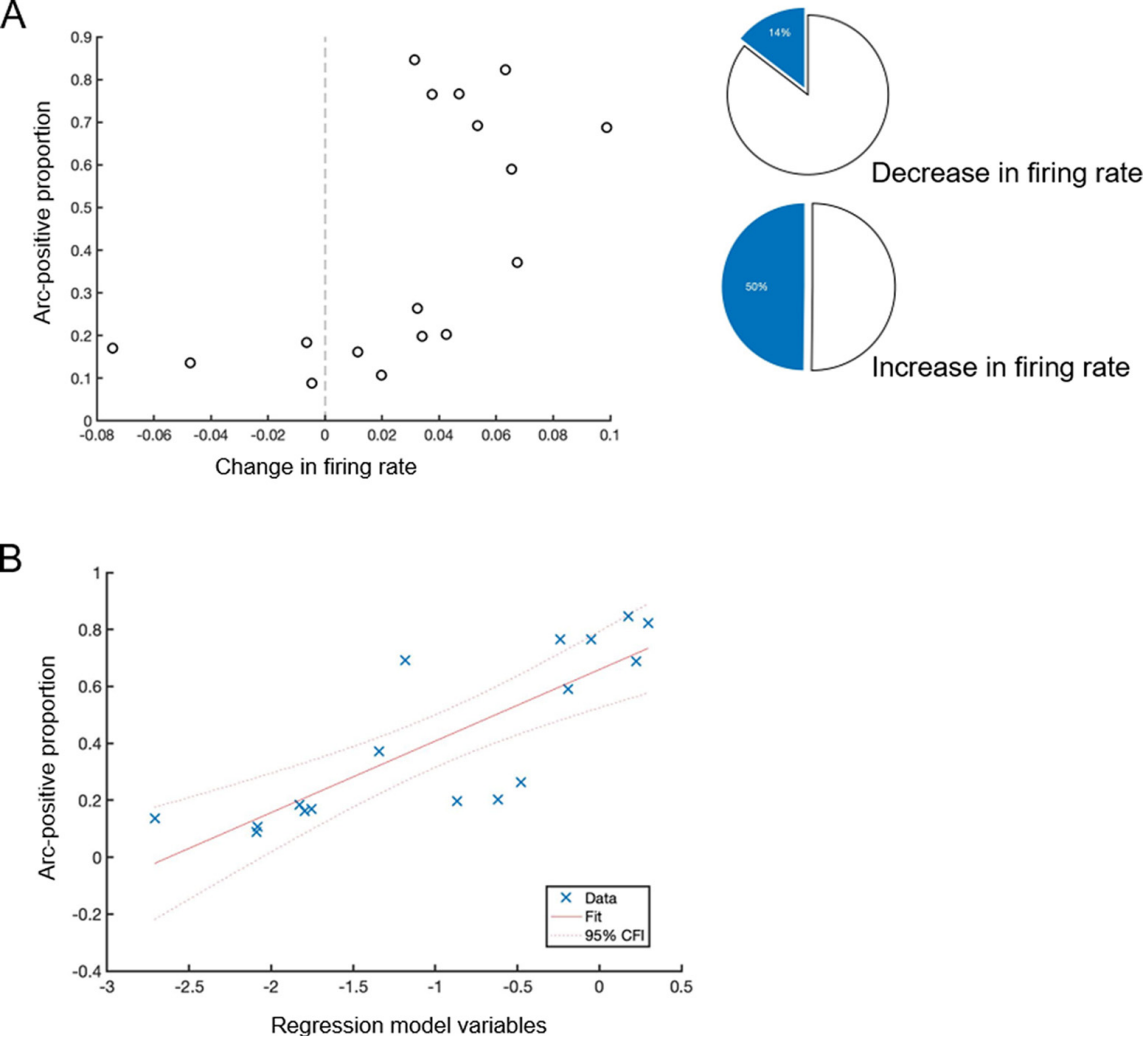
Arc expression and neuronal activity. ***A***, Graph showing change in firing rate after 4BF versus percentage of Arc expression in the culture. Side panel pie charts: a reduction in firing rate is associated with a low level of Arc expression (14%), while an increase in firing rate is associated with a higher level of Arc expression (50%). This was found from a total of *n* = 17 cultures. ***B***, Linear regression model with stepwise addition of factors CI (STTC values), firing rate, state of refinement (binary factor). It was found that the final STTC value together with the state of refinement are most able to explain the level of Arc expression after 4BF.

To determine how refinement is related to Arc expression, the percentage of Arc-positive neurons was determined by immunofluorescent staining and categorically compared as in Figure 5. Our results show that cultures expressing high levels of Arc are more likely to have undergone refinement. To test this observation more robustly, a linear regression model was used to fit the data, with the final Arc expression percentage as the outcome. Of all the input variables (including firing rate before and after 4BF, change in firing rate, connectivity indices before and after 4BF, change in connectivity index, and whether the network has undergone refinement), it was found that the final average network CI after 4BF treatment and refinement status can best predict the level of Arc expression in the network (Fig. 5*B*; Table 1). A total of *n* = 17 cultures was used for this analysis, with staining for both NeuN and Arc.

**Table 1:** Linear regression model variables and statistics

Linear regression model					
Arc-positive percentage = 1 – 0.269 × final CI + 0.250 × refinement status					
					
		Estimate	SE	*T*	*P*
Final CI	−0.0269	0.00608	−4.43	0.000575	
Refinement status	0.2499	0.0876	2.85	0.0128	
					
ANOVA					
	DF	SS	MS	*F*	*P*
Regression	2	0.912	0.456	14.5	0.000392
Error	14	0.441	0.0316		
Total	16	1.35	0.0846		
*R*^2^_adjusted_ = 0.627					

Stepwise linear regression was used to determine variables that best explain the Arc-positive percentage (level of Arc expression in each culture). Final CI (average STTC) and the refinement status of the culture was found to be the best two variables. SE = standard error; *T* = *t* statistics; *P* = *p* values. Analysis of variance was done for this model with *F*_(2,14)_ = 14.46. DF = degree of freedom; SS = sum squares; MS = mean squares; *F* = *F* statistics; *P* = *p* values. The squared multiple correlation *R*^2^ = 0.627, indicating that 62.7% of the variability in the Arc-positive percentages can explained by changes in Final CI and Refinement status of the cultures.

**Figure 6.**
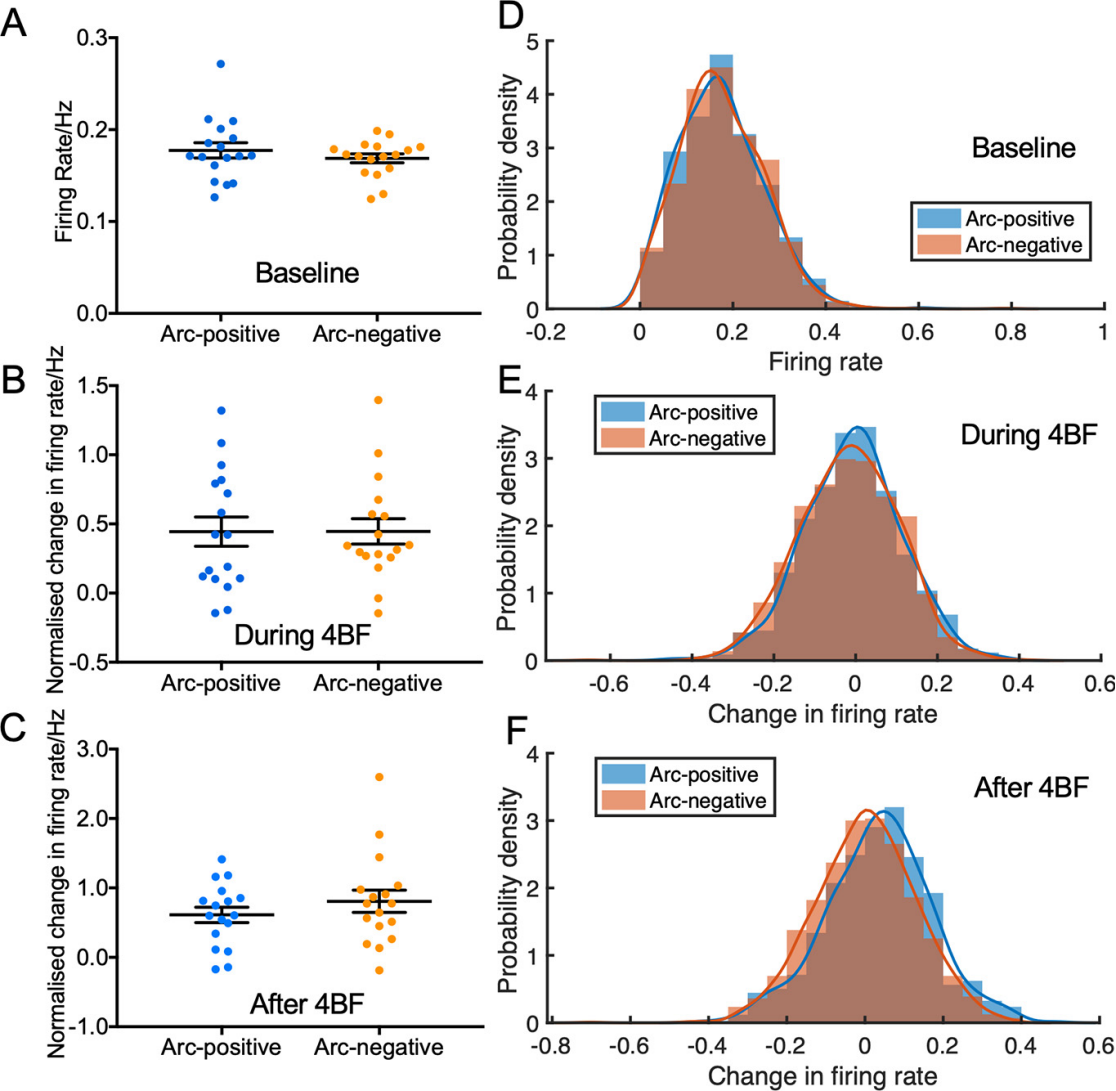
Difference in firing rates between Arc-positive and Arc-negative neurons. ***A***, Firing rate of Arc-positive and Arc-negative neurons in each culture. ***B***, ***C***, Change in firing rate of Arc-positive and Arc-negative neurons in each culture, normalized to baseline, during, and after 4BF treatment (*n* = 17 cultures). ***D***, Firing rate of all Arc-positive (blue) and Arc-negative (orange) neurons across all cultures tested (*n* = 17 cultures). ***E***, ***F***, Change in firing rate of all Arc-positive (blue) and Arc-negative (orange) neurons during and after 4BF, respectively.

Arc is commonly used as a marker of neuronal activity, to determine whether Arc-positive neurons have higher activity than Arc-negative neurons, we specifically examined the firing rates within Arc-positive neurons. Initial analysis of Arc-positive neurons within each network (from a total of *n* = 17 cultures) revealed that there was no difference in firing rates between Arc-positive (both NeuN and Arc-positive) and Arc-negative neurons (NeuN-labeled but Arc-negative; Fig. 6*B*,*C*). However, by examining the firing rate changes in individual neurons, we observe a slight increase in firing rate in Arc-positive neurons after 4BF treatment (Fig. 6*F*).

### Correlated activity between Arc-positive neurons is selectively increased and refined

**Figure 7.**
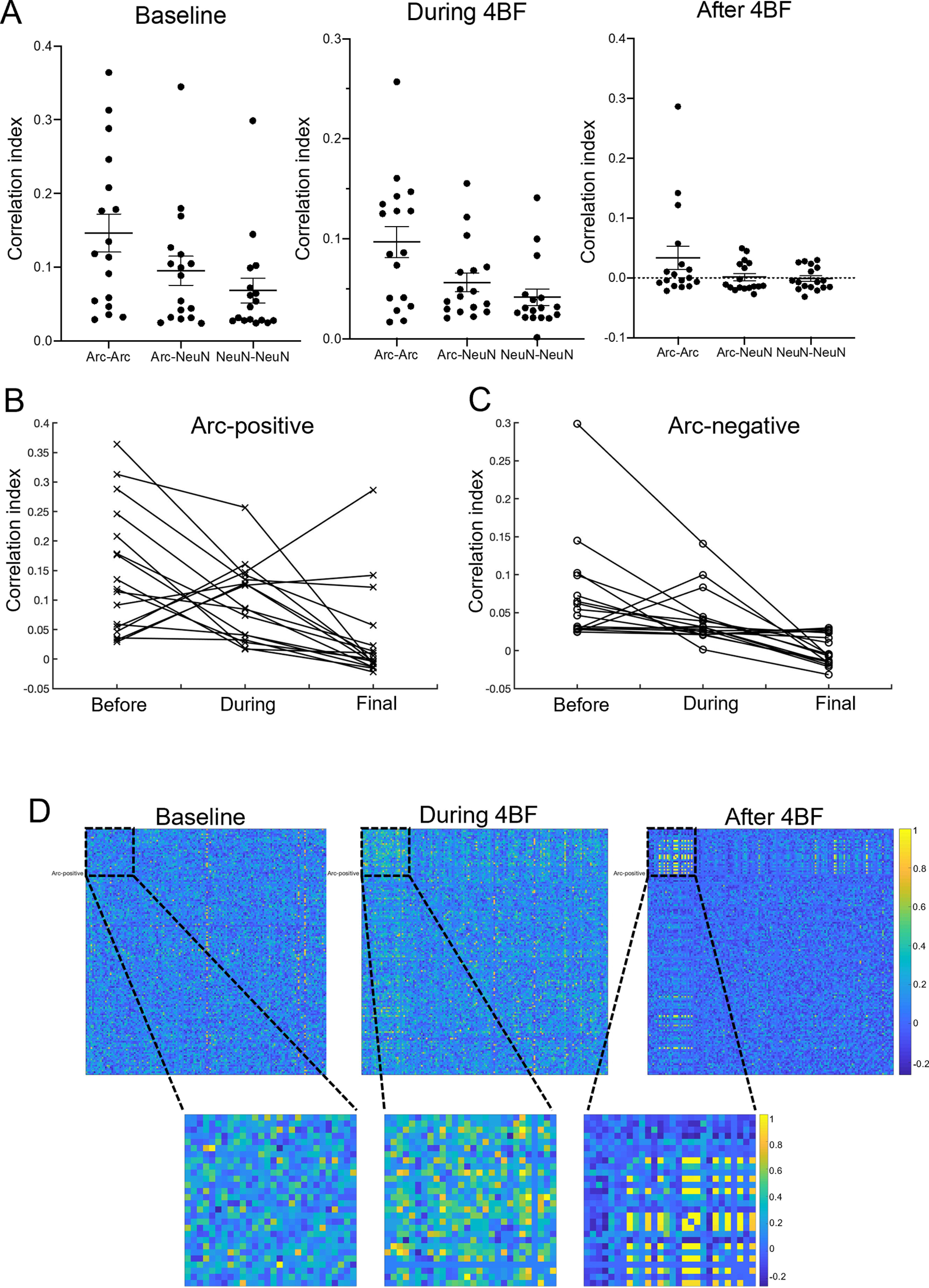
Difference in correlated activity between Arc-positive and Arc-negative neurons. ***A***, CI between Arc-positive neurons in each culture and those that do not have Arc. Arc-positive neurons in each culture exhibit higher CI than other non-Arc neurons at baseline, during, and also after 4BF treatment. Mean CI between Arc-Arc neurons at baseline = 0.147, SEM = 0.0258. Mean CI between Arc-NeuN neurons at baseline = 0.0954, SEM = 0.0197. Mean CI between NeuN-NeuN neurons at baseline = 0.0686, SEM = 0.0166. Mean CI between Arc-Arc neurons during 4BF = 0.0967, SEM = 0.0157. Mean CI between Arc-NeuN neurons during 4BF = 0.0562, SEM = 0.00928. Mean CI between NeuN-NeuN neurons during 4BF = 0.0415, SEM = 0.00844. Mean CI between of Arc-Arc neurons after 4BF = 0.0336, SEM = 0.0195. Mean CI between of Arc-NeuN neurons after 4BF = 0.00108, SEM = 0.00598. Mean CI between of NeuN-NeuN neurons after 4BF = −0.00127, SEM = 0.00474. A total of *n* = 17 cultures were examined. ***B***, Some of the Arc-positive neuronal population increased in CI after 4BF. These were cultures which exhibited lower initial CI. ***C***, Such increase is not seen in the Arc-negative population. ***D***, Representative adjacency matrices of a culture at baseline, during, and after 4BF treatment, showing selective increase and refinement in the Arc-positive neurons.

To determine whether there is any relationship between expression of Arc and neuronal activity correlations, we specifically examined the CI of Arc-positive neurons in each culture and those that do not express Arc (only labeled with the neuronal marker NeuN). Results indicate that Arc-positive neurons in each culture exhibit higher connectivity than other non-Arc neurons (Fig. 7*A*,*B*). This is the case at baseline, during, and also after 4BF treatment (Fig. 7*A*). Cultures tend to have an overall reduction in CI after 4BF treatment (Fig. 7*C*). By separating the analysis into Arc and non-Arc neurons, the Arc-positive neuronal population showed increased CIs after 4BF (Fig. 7*B*). Specifically, some cultures which exhibited lower initial CI increased in CI only in the Arc-positive population but not in the Arc negative population (Fig. 7*B*,*C*). This selective increase can also be seen from the adjacency matrix, a representative one being shown in Figure 7*D*.

**Figure 8.**
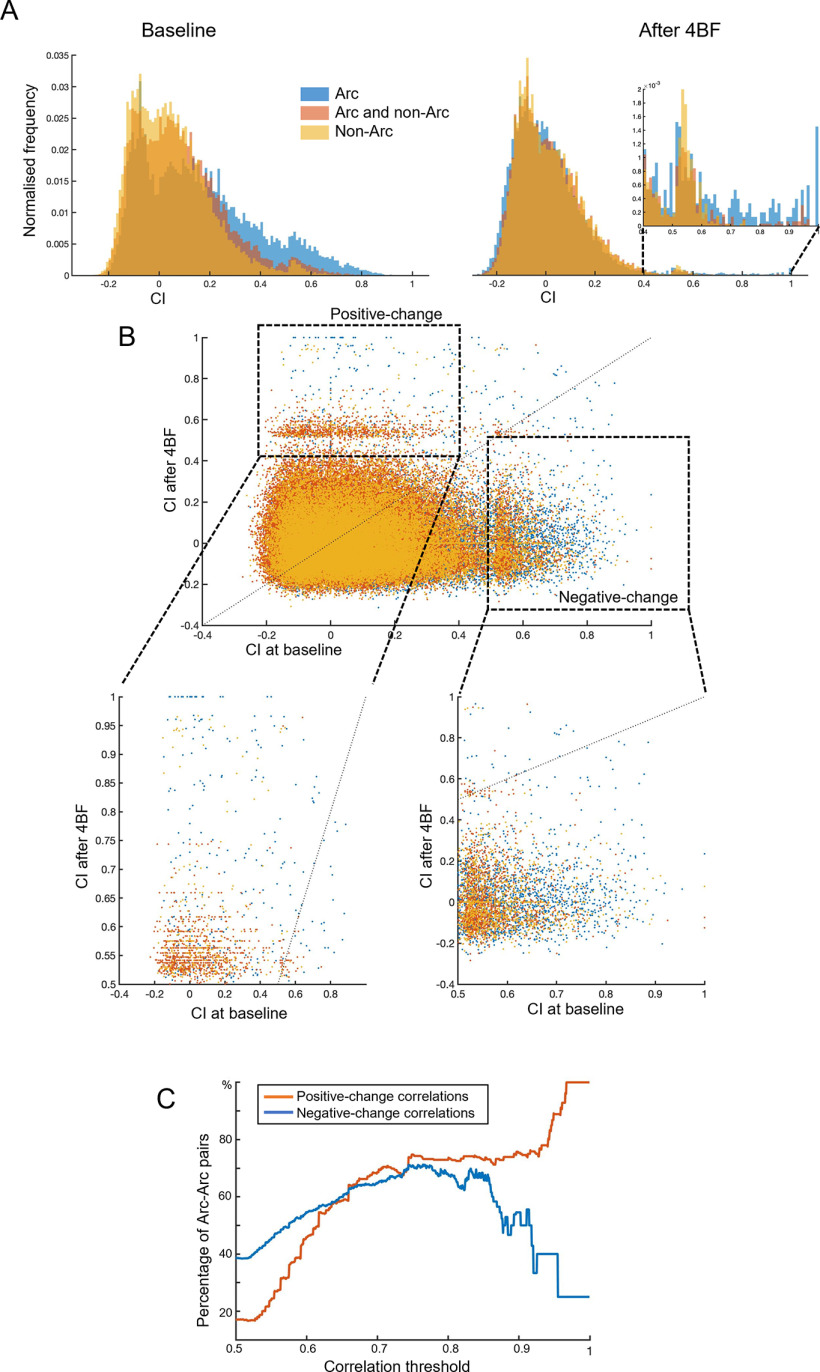
Difference in CI between neuronArc-positive neurons and those that do not. ***A***, Pairs of neurons are grouped into: Arc and Arc (blue), Arc and non-Arc (orange), and non-Arc and non-Arc (yellow). The distribution of CI according to their groups (labeled in different colors) are shown in the histograms, with the left panel being the CI between all pairs of neurons at baseline and the right panel being the CI between all pairs of neurons after 4BF treatment. The *x*-axis represents the range of CI, which are mostly between the values of −0.2 to 1. The *y*-axis represents the frequency at which each CI interval occurs, with a peak at around zero. This indicates that most of the CI are of low values. The inset on the left panel is a zoomed-in view of the higher CI between 0.4 and 1. This shows that the highest CI among pairs of neurons after 4BF treatment occur between Arc-positive and Arc-positive neurons. ***B***, Scatter plot of CI before 4BF (at baseline) and after 4BF treatment. Left dotted box indicates CI that are of low values initially that have increased in value after 4BF treatment (positive-change correlations), whereas right dotted box marks out CI that are initially of high values that have decreased after 4BF treatment (negative-change correlations). The lower left and right panels are zoomed in views of positive-change and negative-change correlations, respectively. ***C***, The percentage of Arc-positive to Arc-positive pairs is determined by counting the numbers of Arc-Arc pairs over the total number of neuronal pairs in the culture at each correlation threshold. Positive-change correlations are those that have initial CI between the values of 0 and 0.5 and final values between 0.5 and 1, while negative-change correlations are defined as those that are initially between 0 and 0.5 and have final values between 0.5 and 1. The *x*-axis represents the range of correlation thresholds above which the percentage Arc-Arc pairs are evaluated; for positive-change correlations, the threshold is based on the final CI while for negative-change correlations the thresholds are determined based on the initial CI.

To investigate the effects of 4BF on neuronal activity correlations especially in the Arc-positive neuronal population, all individual correlations between neuronal pairs were examined. These correlations can be grouped into correlations within the Arc-positive populations (that is the correlations between two Arc-positive neurons), correlations between Arc-positive neurons and Arc-negative neurons, and finally correlations between the Arc-negative neurons. The CI of these groups can be represented with a histogram as shown in Figure 8*A*. It can be seen that the CI among Arc-positive neurons are higher than the CI between Arc and non-Arc and within non-Arc neurons at baseline. This means that neurons that initially had high CI are more likely to express Arc after the 4BF treatment. Subsequently, after 4BF treatment, the overall CI for all groups were reduced, but there remained a small number of correlations with high CI, especially from the Arc-Arc group (Fig. 8*A*, right panel inset). To examine the change for each correlation after 4BF treatment, the initial CI at baseline was plotted against the final CI after 4BF (Fig. 8*B*). This analysis revealed that many correlations have decreased CI (“negative-change correlations”) after 4BF treatment (Fig. 8*B*, negative-change correlations box, right panel inset). However, a subpopulation of correlations showed increase in CI (“positive-change correlations”) after 4BF treatment (Fig. 8*B*, positive-change correlations box, left panel inset). To determine the proportion of Arc-Arc correlations among the positive-change versus negative-change correlations, the percentages of Arc-Arc correlations were calculated as a function of the final (for positive-change) or initial (for negative-change) CI (labeled here as the correlation threshold), as seen in Figure 8*C*. This reveals that there is a large proportion of Arc-Arc correlations with high CI after the 4BF treatment, and these correlations were initially low. This therefore supports the hypothesis that a subset of correlations between Arc-positive neurons are selectively increased after 4BF treatment.

### Arc-positive neurons are located physically closer to each other

**Figure 9.**
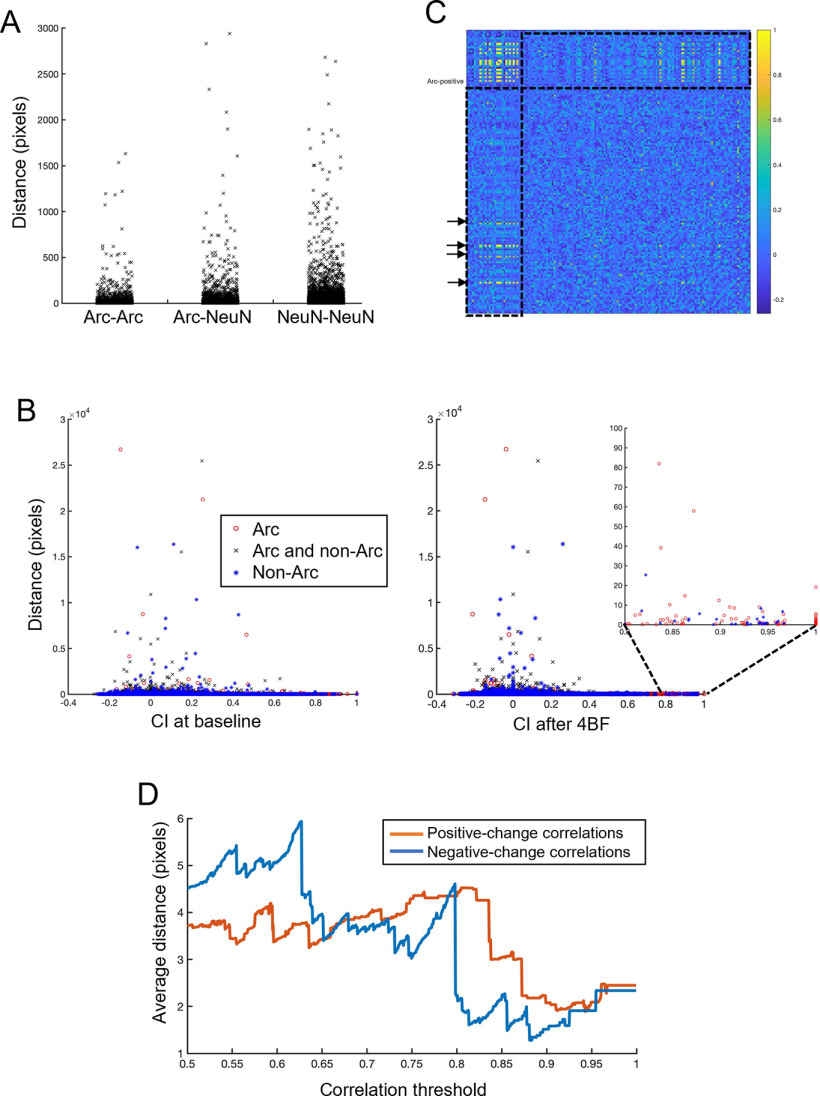
Distance measures of different types of neuronal pairs. ***A***, Distances between pairs of neurons for each type of neuronal pair. Arc-Arc: Arc-positive neurons and Arc-positive neurons. Arc-NeuN: Arc-positive neurons and Arc-negative (non-Arc) neurons. NeuN-NeuN: Arc-negative neurons and Arc-negative neurons. ***B***, Distances between pairs of neurons versus CI at baseline (left panel) and after 4BF (right panel). Different types of neuronal pairs marked with separate types of markers (see legend). Zoomed-in inset on the right panel shows the same graph with CI between 0.8 and 1. ***C***, Representative adjacency matrix after 4BF treatment (as in Fig. 7*D*). Dotted boxes show the CI profile between Arc-positive and Arc-negative neurons, with arrows indicating high CI. ***D***, Positive-change correlations are those that have initial CI between the values of 0 and 0.5 and final values between 0.5 and 1 while negative-change correlations are defined as those with CI that are initially between 0 and 0.5 and final values between 0.5 and 1. The *x*-axis represents the range of correlation thresholds above which the percentage Arc-Arc neuron pairs are evaluated; for positive-change correlations, the threshold is based on the final CI while for negative-change correlations the thresholds are determined based on the initial CI. The *y*-axis shows values of average distances of all neuronal pairs above the threshold value in *x*. Distance measured in pixels, with each pixel corresponding to 0.8 μm.

To examine whether there is any relationship between the status of Arc expression and the physical distances between pairs of neurons, the coordinates of the neurons in each culture were obtained. Distances between each pair of neurons were calculated as described in Materials and Methods. Figure 9*A* shows the values of distances between pairs of neurons according to their Arc expression status. We found that the distances between Arc-positive neurons are generally shorter than that between Arc and non-Arc and also between the non-Arc neuron pairs. This means that Arc-positive neurons are likely to be closely located in the vicinity of each other. To determine whether there is any correlation between the CI and physical distance between each pair of neurons, we examined the association between distances and CI of each neuronal pair both at baseline (Fig. 9*B*, left panel) and after 4BF (Fig. 9*B*, right panel). 4BF does not significantly change the distribution of distance versus CI of the neuronal pairs. Most of the distances are short-ranged, with a few long-ranged ones that extend up to 2.4 mm. These long-range distances tend to be associated with CI of low values around zero. This is the case both at baseline and also after 4BF treatment. The zoomed-in inset on the right panel of Figure 9*B* shows that correlations with high CI after 4BF treatment are likely to have short-range distances between pairs of neurons of which at least one is Arc-positive. In Figure 9*C*, high CI between Arc and non-Arc neurons marked out with arrows. This distance analysis shows that these correlations that are marked out likely represent neuronal pairs that are in close physical proximity to each other. To determine whether there is any difference in physical distance between positive-change versus negative-change correlations, a similar analysis was done as in the previous section. There is a general down sloping trend for both types of connections, showing that larger changes in CI (both positive and negative) tend to be short ranged (Fig. 9*D*).

### Arc-positive cells are mostly a subset of c-Fos cells

**Figure 10.**
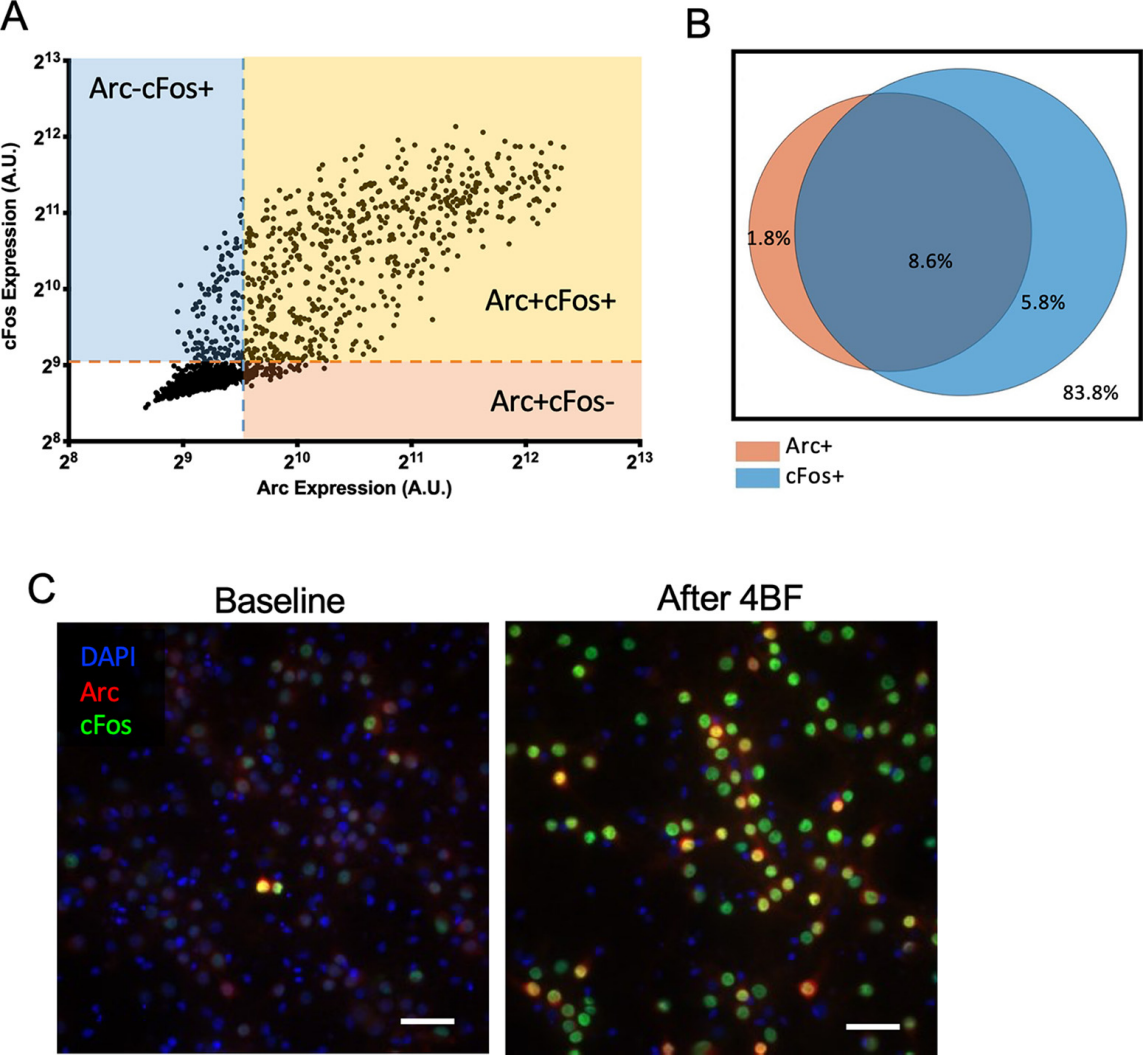
Arc and c-Fos expression after 4BF. ***A***, Expression of Arc and c-Fos in individual neurons from a single frame. This shows the majority of the cells are both Arc and c-Fos-positive, with a good number of c-Fos only expressing cells but few Arc only expressing neurons. ***B***, Pie chart with Arc and/or c-Fos-positive percentages after 4BF treatment. ***C***, Arc and c-Fos expression at baseline and after 4BF treatment.

To investigate the effects of 4BF treatment on the expression of both Arc and c-Fos *in vitro*, the same drug treatment protocol was used and the expression levels of both these proteins were assessed simultaneously using immunofluorescence staining. By colabeling both Arc and c-Fos, it was found that most of the Arc-expressing cells were also c-Fos-positive. This was done in a total of *n* = 11 cultures. Thresholds for detection of positive expression were computed as described in Materials and Methods, and the numbers of Arc and/or c-Fos-positive neurons were quantified and their proportions determined across these eleven cultures. The majority of neurons were positive for both Arc and c-Fos, with some that were only positive for c-Fos and only very few cells that were only positive for Arc (Fig. 10*A*). The pie chart in Figure 10*B* shows that an average of 16.2% of all cells in the culture express Arc and/or c-Fos after 4BF treatment. Among the Arc-positive neurons, 82.7% of these are also c-Fos-positive (Fig. 10*B*).

### Arc and c-Fos expression as a combinatorial code for correlation strength pruning

**Figure 11.**
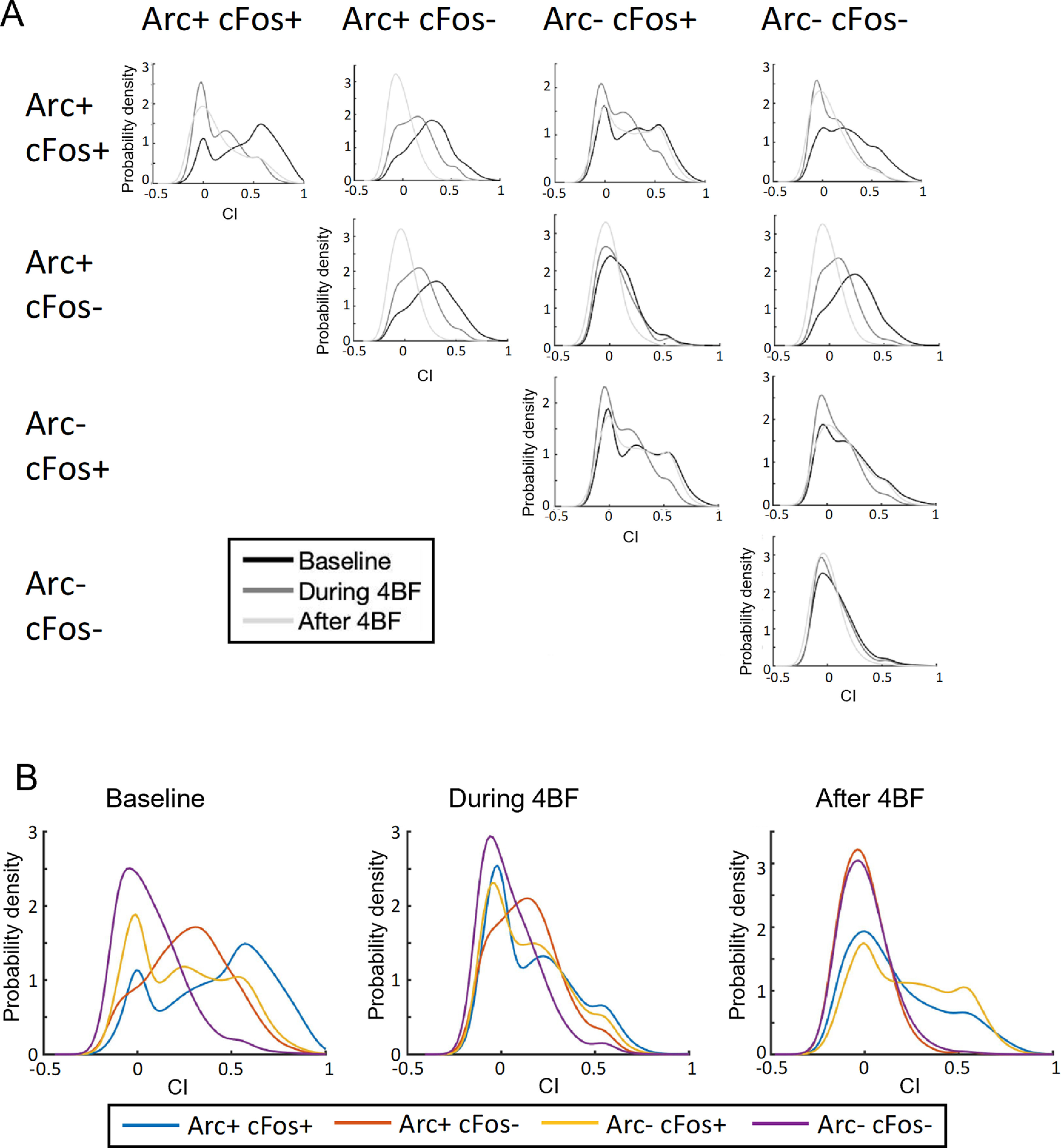
Probability density plots of CI distributions of different populations of neurons (*x*-axis represents the connection strengths and *y*-axis represents frequency of the strengths for all plots). ***A***, Probability density plots of CI distributions between different groups of neurons that express Arc and/or c-Fos or none of these markers. Given that there are four groups of cells depending on the expression status (as listed in the top row and similarly in the first column), there are 10 different sets of connections between these four groups. The CIs between these 10 sets are nonoverlapping and are represented in the subplots I–X. The row and column headers are labeled with the identity of the groups of the neuronal pair. ***B***, The intragroup pairs (subplots along the diagonal, I, V, VIII, and X) are extracted and plotted using different colors on the same graph for baseline, during 4BF treatment, and after 4BF treatment.

Based on the expression profile of Arc and c-Fos, the cells were grouped into Arc**+**cFos**+**, Arc**+**cFos**−**, Arc**−**cFos**+**, and Arc**−**cFos**−**. The intra group and across group correlations in activity between pairs of neurons can thus be classified into 10 different sets. To examine the change across time after 4BF treatment on each set of correlations, probability density distributions of each set of correlations were plotted using a nonkernel density estimation method (Fig. 11*A*). The intragroup correlation sets (Fig. 11*A*, subplots I, V, VIII, X) were also plotted on the same graphs in Figure 11*B* to highlight the differences across different intra-group sets. Results show that double positive neuronal correlations were the strongest before 4BF treatment (Fig. 11*B*, left panel). The correlations between neurons expressing only Arc but not c-Fos to other neurons were specifically downregulated (Fig. 11*A*, subplots II, V, VII,*B*). Interestingly, the c-Fos-only correlations were not changed much after 4BF (Fig. 10*A*, subplots III, VI, VIII, IX). These results support the hypothesis that there is a combinatorial code by the expression of Arc and/or c-Fos in association with changes in correlated activity between these neurons.

## Discussion

We have made use of pharmacological treatment by 4BF on hippocampal neuronal cells, which have been established in previous studies to induce LTP, to show that there is a global reduction in network neuronal activity correlations while a subset of correlations between some of the neurons showed a selective increase. This refinement effect correlates specifically with Arc expression in the network, with Arc-positive neurons demonstrating increase correlated activity while non-Arc neurons demonstrating a decrease in correlated activity. The expression patterns of IEGs Arc and c-Fos were shown to be mostly overlapping, but Arc is more selectively expressed than c-Fos, after induction of chemical LTP by 4BF. The expression status of Arc and c-Fos in the neurons is associated with distinct variations in correlation strength changes before and after 4BF treatment.

### Correlated activity and functional connectivity

The overall goal of this study was to correlate expression of the two IEGs Arc and c-Fos in individual neurons with changes in activity and functional connectivity induced by chemical LTP. Changes in correlated activity between a pair of neurons is indicative of alterations in functional connectivity. However, it does not imply that the synaptic connections between these two neurons have been altered. In fact, it is possible that the two neurons are not synaptically connected, but both receive inputs from the same subset of neurons and their functional connectivity with this subset was altered in the same direction. Given the propensity of these networks of cultured neurons for highly synchronized activity (Dranias et al., 2013), this is not an unlikely scenario. Whereas no firm conclusions can be made regarding changes in functional connectivity for specific neuron pairs, our finding that the subset of Arc-positive neurons displayed a selective increase in activity correlation, suggests that functional connectivity was strengthened between the neurons of this subset.

### Association between IEG expression and correlations in activity

This study aims to relate neuronal activity-dependent expression of the IEGs Arc and c-Fos on the level of individual cells to changes in neuronal activity correlations in the network. We have provided an interesting perspective of the relationship between correlated neuronal activity in a population of cells and the expression of two IEGs, Arc and cFos, on the individual cellular level. However, this work is mostly correlative as we have not determined the mechanism underlying the changes in correlated firing in the culture. We have made some attempts to address this by studying the effects of Arc knock-down on the correlated activities of the neuronal cultures after 4BF. This was done using a previously validated short hairpin RNA (shRNA) construct targeting the coding region of Arc, which has been shown to be able to suppress Arc induction following 4BF treatment (Leung et al., 2019). However, the introduction of two viral vectors to the neurons at high MOI resulted in significant cell death. We therefore did not include these networks for further analysis. We believe that the effects of IEG manipulation on functional connectivity changes is an important next step to further probe the causative relationships that we have observed. Another limitation of our study is that LTP was not measured directly as a result of 4BF. Although 4BF has been previously used to induce LTP, the establishment of LTP in the neuronal network was not empirically determined in our cultures. Our results showed that 4BF resulted in a global reduction of correlated neuronal activity, which seems counterintuitive to effects of LTP. However, we propose that the observed refinement effect may be a network level response in aiding LTP of specific connections, as more contrast is provided to the connections which are strengthened. This effect of network refinement is discussed in more details in a subsequent section.

### Potential sources of error and caveats

In our study, we have identified neurons by NeuN labeling but have not distinguished excitatory projection neurons and inhibitory interneurons. Therefore, differences in the GABAergic interneuronal content in the cultures could contribute in part to the interculture variability in the response to 4BF. We have included visualization of individual datapoints for each culture and showed that the effects we have reported can be distinguished on top of the interculture variability seen. On analysis of correlation between individual pairs of neurons, the CI was computed based on both positive as well as negative correlations to obtain undirected networks, which was an attempt to account for the inhibitory connections. The inhibitory neuron content, however, is an interesting aspect for further analysis in future studies. Another source of error could arise from calcium imaging. The parameters selected for our experiments balances the trade-offs between signal strength and photobleaching. GCaMP6m has a 94% somatic action potential detection efficiency at a 1% false positive rate (Chen et al., 2013). Thus, there could be an underestimation of the number of actual action potentials. Third, the expression of IEGs Arc and c-Fos was assayed at a fixed time point after 4BF treatment. We have chosen this time point based on the peak of IEG expression after 4BF, but this only represents a snapshot in the expression dynamics after neuronal activation. Additional experiments could make use of real-time expression reporters of IEGs to better characterize the relationship between the ongoing level of IEG expression and correlations in activity of the neuronal network. Finally, we have examined correlated neuronal activity without mapping the actual physical synaptic neuronal connections.

### Network refinement as an emergent network property after chemical LTP

The potentiation effects of LTP have been well-studied on the cellular level, with the measurable end result usually being increases in EPSPs or changes in spine size. However, few studies have investigated the effects of changes in neuronal firing behavior (Marder and Buonomano, 2004). One such study by Chiappalone et al. (2008) using neuronal cultures grown on MEAs found that a tetanus stimulus can induce an overall decrease in evoked network activity, although the same stimulus has been previously shown to induce potentiation of specific pathways (Jimbo et al., 1999). This is consistent with the effects of refinement in correlated activity after 4BF treatment. It has long been thought that plasticity mechanisms cause networks to destabilize (Abbott and Nelson, 2000), and many cellular and network mechanisms, generally termed as homeostatic plasticity, have been discovered to push their state back to baseline (Turrigiano, 2012). Among the few cultures that had a decrease in firing rate in response to 4BF, all of them had high network average CI. However, there is a dissociation between the changes in firing rate and correlated activity following 4BF treatment. Although most of the cultures showed an increase in firing rate, there is a decrease in network average CI in all the cultures. Homeostatic synaptic scaling that is required for firing rate modifications in response to prolonged neuronal activity is thought to involve the differential expression of AMPA receptor subunits (Goold and Nicoll, 2010) and a reduction of AMPA receptors at the synapse (Seeburg and Sheng, 2008). It is known that Arc over-expression in hippocampal cultures blocks homeostatic upregulation of AMPA receptors in chronic inactivity (Shepherd et al., 2006). The synaptic scaling that results from chronic activity is commonly observed at 48 h after stimulus onset (Turrigiano et al., 1998). The changes reported here on network neuronal correlations take place in a shorter time frame, ∼6–8 h after 4BF is applied, and therefore the changes in correlated activity may not be a result of the usual mechanisms known in synaptic scaling.

It is known that when LTP reaches saturation, no further learning can occur (Moser et al., 1998). Thus, downregulation of correlated activity in response to prolonged activity is necessary for the network to continue coding useful information. Besides cell autonomous mechanisms of achieving homeostasis, networks are known to maintain stability through coordinated intercellular interactions (Maffei and Fontanini, 2009). One feature worthy of mentioning is the criticality of neuronal networks. Criticality is a network state that maximizes information processing capacities by having high level of responsiveness to external stimuli (Rocha et al., 2018). Using neural networks *in silico*, it has been demonstrated previously that plasticity mechanisms can tune a network to criticality (Rubinov et al., 2011). Effects of 4BF could push the network to criticality and possibly to a supercritical state, where the threshold of activation is high and thus reflects a global reduction in correlated activity. Pharmacological induction, such as the 4BF treatment used here, is often thought to be a strong inducing stimulus that may not be physiologically relevant. However, repeated activation of NMDA receptors is known to be important for memory consolidation (Wittenberg and Tsien, 2002). It has also been shown that during sleep, the majority of cortical synapses are upregulated, supporting the hypothesis that slow oscillations observed during sleep potentiate synapses that were depressed because of persistent activities during the day (Timofeev et al., 2001). The refinement process could act to increase the contrast of significant correlations in the network by modulating network threshold. Refinement of network neuronal correlations that we have shown here in this study is an emergent network property in response to chemical LTP.

### Arc-positive neuronal connections exhibit increased activity correlation changes

IEG expression is often used as a marker of neuronal activity. This is consistent with our findings of increased firing rate changes in Arc-positive neurons. However, this effect on increased firing rate is not uniform across all Arc-positive neurons in a given network. This can be seen from the lack of difference between firing rate changes of Arc-positive and Arc-negative neurons when the analysis was conducted on a per culture level. This means that within each network, the population of Arc-positive neurons have variable changes in firing rate, and not all Arc-positive neurons demonstrate increased neuronal activity. We showed that in networks where the effect of 4BF on network refinement is largest, there is correspondingly a high level of Arc expression. Subsequently, not all the neurons in the network express Arc, although they might participate in the increased network firing. In particular, Arc expression is high in neurons with strongly modulated correlated activity, and especially for positive-change correlations. This is consistent with findings *in vivo* by Mahringer et al. (2019) where they found that neurons undergoing the strongest learning-related changes in activity would exhibit increased levels of IEG expression. Furthermore, subsequent analysis with c-Fos also demonstrated that Arc**+**cFos**−** neurons tend to have negative-change correlations. This contributes to the idea that Arc-positive neurons have increased correlation changes (either increased or decreased) after chemical LTP. Arc may be poised to perform a role in this process, as it has been shown to play a part in both LTP as well as long-term depression (LTD; Bramham et al., 2008). Increased functional-connectivity changes of Arc-positive neurons supports the idea that Arc expression is not primarily a marker of neural activity. The current theory of engram formation assumes that all cells that are more active during encoding go on to express the IEG, subsequently become more connected to each other and form the memory engram. Our results suggest that not all Arc-expressing neurons have an increased firing rate after the stimulus. Neurons that are activated during the process of encoding thus do not undergo uniform increase in correlated activity. Our results show that selective neuronal pairs particularly within the IEG-expressing population demonstrate increased correlated activity, with other pairs showing decreased correlated activity. This refinement effect is particularly apparent within the Arc-expressing neuronal population.

### Expression of Arc may depend on network architecture

We have shown that there is a negative correlation between physical distance of neuron pairs and their functional connectivity. Correspondingly, connections that exhibit large activity correlation changes after 4BF treatment tend to have closer physical proximity and involve at least one Arc-positive neuron. This relationship between correlated activity and anatomic distance is consistent with previous reports of findings on the brain level. For instance, fMRI studies have been shown that the functional connectivity is stronger between regions with shorter physical distance and conversely, correlated activity decreases as the physical distance between brain regions increases (Salvador et al., 2005; Bellec et al., 2006). This pattern of functional connectivity variation with physical distance aims to minimize wiring cost and thus follows economical principle in circuit organization. Such a pattern of activity correlations may also be at play *in vitro* for individual connections between neurons. 4BF acts to increase synchronized bursting of the network, however, only a selective subset of neurons express IEGs such as Arc. We have shown here that these selected subsets tend to be located physically closer to each other. The expression of Arc, therefore, may depend on the architecture of the network. Preexisting short-range connections could undergo selective increase in correlated activity.

### Arc and/or c-Fos expression correlates with distinct changes in neuronal correlations

Our results showed that Arc and c-Fos are expressed in subsets of neurons after 4BF induction, and large correlated activity changes are likely to happen among Arc-positive neurons. Further analysis of the costaining with c-Fos reveals that correlated activity within the Arc**+**cFos**−** subgroup of neurons are mostly decreased. On the other hand, there is a high degree of correlated activity among the double-positive (Arc**+**cFos**+**) neurons, the Arc**−**cFos**+** neurons and between these two groups. Arc and c-Fos have been shown to act in combination to specify whether hippocampal neurons are involved in memory reactivation using a novel environment exposure protocol in mice (Jaeger et al., 2018). In their experiments, both Arc and c-Fos are expressed abundantly 1-h after novel environment exposure, and highly overlap, but only Arc persists through 5 h after the exposure. As such, doubly positive Arc**+**cFos**+** neurons are reactivated or newly activated on re-exposure to the same context, whereas Arc**+**cFos**−** neurons are not reactivated on exposure to a new context. Our results show that Arc expression in neurons is correlated with presence of strongly modulated changes in CI. The concurrent presence of c-Fos in these neurons correlate with the direction of the modulation of changes in CI. This is in line with the hypothesis that expression of Arc and c-Fos may act as a combinatorial code for distinguishing the subpopulations of neurons that undergo different types of activity-dependent plasticity, though confirmation of this hypothesis would require additional experiments to demonstrate the causality involved in this relationship.

### Dynamic interplay between network activity, IEG expression, and plasticity

Conventionally, studies of neuronal activity and IEG expression are mostly based on the single cell level whereas network activity is recorded on the neuronal population level. Our work here correlates the expression of the two IEGs Arc and c-Fos in individual neurons with changes in activity and functional connectivity induced by chemical LTP. Some others like Mahringer et al. (2019) have examined the expression of IEGs *in vivo* and linked it to the activity of neurons during specific tasks, which has the benefit of relating individual neuronal responses to specific stimuli presented to the animal. We believe that our study has added to the idea that there is a dynamic interplay between IEG expression and neuronal activity in the network. To further probe the links between IEG expression and connectivity changes, specific stimulus patters selectively activating subsets of neurons could be used, for instance using optogenetic methods. This would provide a direct assessment of the causal relationship between changes in network activity and molecular changes within individual cells, and thereby linking IEG expression, synaptic plasticity, and network connectivity.

In summary, we investigated the network changes that can occur after induction of LTP *in vitro* and found that chemical LTP induction by 4BF resulted in an emergent effect of refinement of the correlated neuronal activities. Arc expression correlates with effects of 4BF, particularly on network refinement, and Arc-positive neurons exhibit more changes in correlated activity after 4BF. We also found that the architecture of the network may affect Arc expression as Arc-positive neurons are located closer to each other in the network. In terms of the expression of IEGs Arc and c-Fos, we found that Arc is more selectively expressed after 4BF. The differential expression of these two IEGs were associated with changes in correlated neuronal activity. With these results we have uncovered some interesting and important links between correlated neuronal activity, and IEG expression.

## Synthesis

Reviewing Editor: Kristen Harris, The University of Texas at Austin Decisions are customarily a result of the Reviewing Editor and the peer reviewers coming together and discussing their recommendations until a consensus is reached. When revisions are invited, a fact-based synthesis statement explaining their decision and outlining what is needed to prepare a revision will be listed below. The following reviewer(s) agreed to reveal their identity: Oswald Steward, Clive Braham.

The authors have made improvements to the paper with the addition of methodological experimental details. However, the authors fail to address the key issues raised in the reviews related to invalid assumptions, use of terminology that does not accurately reflect what is being measured, and lack of critical thinking. Without thorough revision, this paper will not advance understanding.

Title:

Starting from the title, the authors are attempting to draw broad conclusions regarding engrams, LTP, LTD, connections, and neuronal circuits that depend on several faulty theoretical constructs. The authors need to step back from their lofty thinking and re-write the paper with accurate descriptions of what is being measured rather than their conclusions based on concepts of synaptic physiology, learning and memory. First, the title mis-uses terminology. There are no “engrams” in a culture dish of neurons. The use of engram in the title is misleading as it refers to memory. We suggest making the title more specific such as: “Selective increase of functional network activity in Arc-positive neurons after chemically induced long-term potentiation in cultured hippocampal neurons"

Throughout the Manuscript:

The terms “connectivity and circuit” are used in ways that are not linked to actual biology, leading to conclusions that are not based on the data. The solution is to re-write the paper using terms that accurately reflect what is being measured, for example: “correlated activity” rather than “connectivity", “increases vs. decreases in correlated activity” rather than “potentiated vs. depressed connections”.

As used here, the terms are multiple order theoretical constructs based on weak assumptions. The authors apparently assume that their measure of correlated activity is a first order assumption based on the conversion of calcium imaging data to neuronal firing. The use of the term connectivity or circuits in this context assumes that this correlated activity demonstrates that one neuron is connected to another and drives the target neuron’s activity. There are obvious alternatives including that the neurons whose activity is correlated are activated by some other neuron and not connected with one another at all. An analogy might be a wind turbine farm where there is correlated activity due to the wind without any connection between individual turbines. The paper does not include any direct assessment of connections at all and use of this term is the first step in faulty reasoning.

The weak reasoning is amplified when the authors refer to “potentiated connections” and “depressed connections", with the implication that these relate to LTP and LTD studied in defined pathways. The same applies to the phrase “refinement of network connectivity”.

In our opinion, the term “circuit” is also misused. In neuroscience, circuits are defined sets of connections between neuronal populations and brain structures. In the absence of evidence to the contrary, connections between neurons growing in culture are random and in no way like organized neural circuits in the brain.

Last sentence of the Abstract: Suggest removing “on the circuit level” as there is no defined circuitry.

The Discussion takes all of this issue even further by attempting to relate the data from neurons in culture with synaptic plasticity in vivo, neuronal circuits in the brain, learning and memory, etc. All of which is complete speculation.

Nevertheless, we expect that when properly presented and interpreted, the data are of interest. Providing that the Introduction, Methods and Results are linked directly to the actual data, there could be room in the Discussion for nuanced speculation about issues regarding neurons in culture including whether and how correlated activity might indicate connectivity and whether changes in correlated activity might be related to changes in the strength of connections.

Specific Suggestions:

Introduction: Regarding the paper of Jaeger et al, the comment about sustained Arc transcription applies to dentate granule cells. The Arc transcription and mRNA elevation is short-lived in CA1 pyramidal cells.

Discussion: The revised manuscript still indicates a superficial exploration at best of previous studies of co-expression of Arc and c-fos in different settings. The data reported here add little if anything to that story, and most of the Discussion regarding IEG expression could (should) be greatly reduced or deleted.

Discussion: line 605. Regarding Shepherd et al. 2006, note that ectopic expression of Arc in hippocampal neuronal cultures results in global homeostatic downscaling. This effect is present at 16 hours post-transfection. line 645. delete “change"

The heading: “Arc positive cells exhibit a higher firing rate” overstates the very tiny difference that probably has little if any biological significance, especially given the high variability across the population.

Figure 5B presents data for two arbitrary categories (with and without refinement). However, the category “With refinement” has two distinct populations (n=6 above 0.6 and n=4 below 0.4 at the same value as the “No refinement” category). These outcomes suggest that the division into these binary categories is not meaningful. The non-categorical analyses make fewer assumptions and are more informative.

Figure 7 The significance of the results presented in figure 7 is questionable because the findings are seen in only some cultures. Without some understanding of the source of this variability, any overarching conclusions are un-warranted.

## Author Response

The authors have made improvements to the paper with the addition of methodological experimental details. However, the authors fail to address the key issues raised in the reviews related to invalid assumptions, use of terminology that does not accurately reflect what is being measured, and lack of critical thinking. Without thorough revision, this paper will not advance understanding.

Title:

Starting from the title, the authors are attempting to draw broad conclusions regarding engrams, LTP, LTD, connections, and neuronal circuits that depend on several faulty theoretical constructs. The authors need to step back from their lofty thinking and re-write the paper with accurate descriptions of what is being measured rather than their conclusions based on concepts of synaptic physiology, learning and memory.

First, the title mis-uses terminology. There are no “engrams” in a culture dish of neurons. The use of engram in the title is misleading as it refers to memory. We suggest making the title more specific such as: “Selective increase of functional network activity in Arc-positive neurons after chemically induced long-term potentiation in cultured hippocampal neurons"

The title has been changed. The term “functional connectivity” is clarified in the abstract and is also widely known to specifically refer to correlated activity (eg. See Poli et al 2015, Figure 3). Other instances with the use of the word “connectivity” have been thoroughly changed to “correlated activity”.

Throughout the Manuscript:

The terms “connectivity and circuit” are used in ways that are not linked to actual biology, leading to conclusions that are not based on the data. The solution is to re-write the paper using terms that accurately reflect what is being measured, for example: “correlated activity” rather than “connectivity", “increases vs. decreases in correlated activity” rather than “potentiated vs. depressed connections”.

The terms related to “connectivity” has been changed to “correlated activity”. “Potentiated vs. depressed connections” have been changed too.

As used here, the terms are multiple order theoretical constructs based on weak assumptions. The authors apparently assume that their measure of correlated activity is a first order assumption based on the conversion of calcium imaging data to neuronal firing. The use of the term connectivity or circuits in this context assumes that this correlated activity demonstrates that one neuron is connected to another and drives the target neuron’s activity. There are obvious alternatives including that the neurons whose activity is correlated are activated by some other neuron and not connected with one another at all. An analogy might be a wind turbine farm where there is correlated activity due to the wind without any connection between individual turbines. The paper does not include any direct assessment of connections at all and use of this term is the first step in faulty reasoning.

The terms related to “connectivity” has been changed to “correlated activity”. The fact that actual physical connections between neurons are not measured in this study has also been included within the section under “potential sources of error and caveats”.

The weak reasoning is amplified when the authors refer to “potentiated connections” and “depressed connections", with the implication that these relate to LTP and LTD studied in defined pathways. The same applies to the phrase “refinement of network connectivity”.

The terms involving “potentiated and depressed connections” has been changed to “increased or decreased correlated activity”. “Refinement of network connectivity” has been changed to “refinement in correlated activity”.

In our opinion, the term “circuit” is also misused. In neuroscience, circuits are defined sets of connections between neuronal populations and brain structures. In the absence of evidence to the contrary, connections between neurons growing in culture are random and in no way like organized neural circuits in the brain.

The terms involving “circuit” have been revised.

Last sentence of the Abstract: Suggest removing “on the circuit level” as there is no defined circuitry.

This sentence has been removed.

The Discussion takes all of this issue even further by attempting to relate the data from neurons in culture with synaptic plasticity in vivo, neuronal circuits in the brain, learning and memory, etc. All of which is complete speculation.

Nevertheless, we expect that when properly presented and interpreted, the data are of interest. Providing that the Introduction, Methods and Results are linked directly to the actual data, there could be room in the Discussion for nuanced speculation about issues regarding neurons in culture including whether and how correlated activity might indicate connectivity and whether changes in correlated activity might be related to changes in the strength of connections.

We have added a section (highlighted in yellow) discussing the problems associated with the interpretation of changes in correlated neuronal activity in terms of alterations in functional connectivity.

Specific Suggestions:

Introduction: Regarding the paper of Jaeger et al, the comment about sustained Arc transcription applies to dentate granule cells. The Arc transcription and mRNA elevation is short-lived in CA1 pyramidal cells.

Edited to include the location of the neurons.

Discussion: The revised manuscript still indicates a superficial exploration at best of previous studies of co-expression of Arc and c-fos in different settings. The data reported here add little if anything to that story, and most of the Discussion regarding IEG expression could (should) be greatly reduced or deleted.

The Discussion regarding IEG expression in vivo has been condensed.

Discussion: line 605. Regarding Shepherd et al. 2006, note that ectopic expression of Arc in hippocampal neuronal cultures results in global homeostatic downscaling. This effect is present at 16 hours post-transfection.

Changed to “It is known that Arc over-expression in hippocampal cultures blocks homeostatic upregulation of AMPA receptors in chronic inactivity.”

line 645. delete “change” deleted

The heading: “Arc positive cells exhibit a higher firing rate” overstates the very tiny difference that probably has little if any biological significance, especially given the high variability across the population.

This heading has been deleted, section merged with previous section.

Figure 5B presents data for two arbitrary categories (with and without refinement). However, the category “With refinement” has two distinct populations (n=6 above 0.6 and n=4 below 0.4 at the same value as the “No refinement” category). These outcomes suggest that the division into these binary categories is not meaningful. The non-categorical analyses make fewer assumptions and are more informative.

Fig 5B has been deleted.

Figure 7 The significance of the results presented in figure 7 is questionable because the findings are seen in only some cultures. Without some understanding of the source of this variability, any overarching conclusions are un-warranted.

We have tried to avoid overarching conclusions for this figure.
